# Development of frailty subtypes and their associated risk factors among the community-dwelling elderly population

**DOI:** 10.18632/aging.102671

**Published:** 2020-01-16

**Authors:** Yan Zhang, Xiu-Juan Xu, Ting-Yu Lian, Ling-Feng Huang, Jin-Mei Zeng, Dong-Mei Liang, Ming-Juan Yin, Jing-Xiao Huang, Liang-Chang Xiu, Zu-Wei Yu, Yu-Lian Li, Chen Mao, Jin-Dong Ni

**Affiliations:** 1 Department of Epidemiology and Biostatistics, School of Public Health, Guangdong Medical University, Dongguan 523808, China; 2 Public Health Office, Dalang Town Community Health Service Center, Dongguan 523808, China; 3 Department of Nursing, Dalang Town Community Health Service Center, Dongguan 523808, China; 4 Department of Epidemiology, School of Public Health, Southern Medical University, Guangzhou 510515, China

**Keywords:** frailty, subtypes, profiles, older people, risk factors

## Abstract

In order to explore frailty subtypes and find their associated risk factors, we conducted cross-sectional surveys of 5,341 seniors aged 60 and over in China using the Frailty Index (FI) scale. We identified four frailty subtypes, namely multi-frail, cognitive and functionally frail, psychologically frail and physiologically frail. Old age and low education level were the common risk factors among the four subtypes. Being widowed, divorced or unmarried was a risk factor for multi-frail, cognitive and functionally frail and psychologically frail, and male sex was a protective factor against cognitive and functionally frail and psychologically frail subtypes. Having a harmonious relationship with family was a protective factor against multi-frail, and fewer visits to the elderly by their children was a risk factor for psychologically frail. Dissatisfaction with their housing was a risk factor for cognitive and functionally frail, psychologically frail and physiologically frail, and a pension being the main source of income was a risk factor for cognitive and functionally frail and psychologically frail. Exercising every day was a protective factor against multi-frail and cognitive and functionally frail, and a lower level of physical activity was a risk factor for all four frailty subtypes. Our findings confirm the heterogeneity of frailty and suggest that different frail elderly individuals need more targeted care interventions.

## INTRODUCTION

With the rapid aging global population, frailty, a multidimensional syndrome associated with subclinical dysfunction and limited physiologic reserve that may affect one or more organs, has received increasing attention [1]. Frailty is common among older people, affecting 7%–12% of adults aged 65 years and older [2], and makes them vulnerable to stressful events and functional deterioration [3, 4]. Frailty can increase the risk of adverse outcomes, such as a decline in functional ability, falls, delirium, etc., and has been identified as a precursor to disability, institutionalization, and mortality in older adults [5, 6]. It also puts increasing pressure on the health care system as frail elderly people are more likely to be hospitalized or to need critical care. In short, frailty severely affects work and quality of life in the elderly, increasing the social and family burden [7]. Thus, frailty should be recognized as a public health priority [8].

Frailty is a heterogeneous condition, which may account for the variations in definitions and assessment methods [9]. Frailty Index (FI), based on a model of accumulation of multi-component deficits, is currently a widely used approach to measure frailty [10, 11]. However, the FI score is a ratio of the number of deficits present to the total number of deficits considered [12], it does not reflect the weight of individual components and it is uncertain how components are clustered [12, 13]. Exploring potential subtypes may improve our understanding of the condition, by explaining the diversity of interactions between different components in elderly people [13, 14]. The use of subtypes is common in other disciplines such as environmental science, social sciences and medical sciences [15–17]. Recently, some subtypes have also been applied in studies of frailty, such as physical frailty [13] and cognitive frailty [18]. However, in these studies, physical functioning and cognitive state were treated as independent components, and the studies did not include other domains, e.g., psychological, social, etc. These domains of frailty must be seen as integrated concepts that could better explain human functioning [19].

Identification of factors associated with frailty is clearly important for developing customized intervention plans for the elderly [20]. Many studies have found some related risk factors of frailty. Chamberlain and colleagues found that some social and behavioral factors, including education, marital status, living arrangements and smoking status, were associated with frailty [11]. Gale et al. found that a high level of loneliness was associated with an increased risk of becoming physically frail [21]. A systematic review concluded that many sociodemographic, physical, biological, lifestyle, and psychological factors showed significant associations with frailty [22]. However, these studies used traditional assessment indicators and standards. Risk factors associated with frailty based on frailty subtypes remain to be studied.

We attempted to bridge this gap in this study using an elderly population in a city in southern China. We applied a frailty assessment tool with multiple functional components but used non-traditional evaluation criteria to: 1) explore subtypes of frailty, and further identify distinct subgroups of subjects; and 2) find factors associated with different frailty subtypes. Our research involved multiple components and explored their potential associations, in order to clarify specific patterns of underlying problems of frail and pre-frail elderly people. A better understanding of factors associated with different subtypes among older people could lead to more targeted population interventions and management. This could prevent or delay disease development, resulting in improved quality of life, and further reduction in health costs.

## RESULTS

### Participant characteristics

Among all 5,341 elderly people surveyed, more than half were women (61.1%), and almost half were 60 to 70-years-old (46.8%). Most (56.9%) of the elderly people had received only primary education. The vast majority were married (74.3%), as shown in Table 1.

**Table 1:** Sociodemographic characteristics of participants.

**Characteristics**	**No. of participants**	**% participants**
Sex*		
Male	2049	38.4
Female	3261	61.1
Age (years old) *		
60~	2501	46.8
70~	1912	35.8
80~	907	17.0
Educational level*		
Illiteracy	1082	20.3
Primary school	3040	56.9
Junior high school and above	1158	21.7
Marital status*		
Married	3971	74.3
Widowed / Divorced / Unmarried	1224	22.9

(N =5341).

* Variables with missing values: Sex (31/5341; 0.6%), Age (21/5341; 0.4%), Educational level (61/5341; 1.1%), and Marital status (146/5341; 2.7%).

### Latent class model fitting results

The study extracted one to seven potential class models, and the fitting results are shown in Table 2. As model classification increased from one to seven, the AIC, BIC, and aBIC continued to decrease, while the values of the Lo–Mendell–Rubin (LMR) test and the bootstrap-based likelihood ratio test (BLRT) reached significant levels (*P* < 0.01) when five categories were retained, and the LMR was no longer significant when the sixth classification was made. According to the LMR and BLRT indicators, the model with five sub-categories was significantly better than the model with four sub-categories, and the model with six sub-categories was not as well fitted as the model with five sub-categories. Therefore, the classification using five latent classes (Class 1, Class 2, Class 3, Class 4, Class 5) was finally selected.

**Table 2:** Fitting statistical results of the latent class model.

**Model**	**K**	**AIC**	**BIC**	**aBIC**	**Entropy**	**LMR**	**BLRT**
1-Class	33	133821.59	134038.84	133933.98	_	_	_
2-Class	67	115664.58	116105.65	115892.75	0.98	<0.001	<0.001
3-Class	101	111917.89	112582.79	112261.84	0.81	0.004	<0.001
4-Class	135	108715.27	109604.00	109175.02	0.85	<0.001	<0.001
5-Class	169	107787.39	108899.94	108362.92	0.83	<0.001	<0.001
6-Class	203	106947.17	108283.56	107638.49	0.83	0.241	<0.001
7-Class	237	106391.29	107951.50	107198.39	0.78	0.0341	<0.001

Abbreviations: AIC, Akaike information criterion; BIC, Bayesian information criterion; aBIC, adjusted Bayesian information criterion; LMR, Lo–Mendell–Rubin; BLRT, bootstrapped likelihood ratio test.

### Naming of latent classes

According to the conditional probability distribution differences and characteristics of 33 items (Supplementary Table 1), each latent class could be named. Compared with the other four classes, class 1 had the highest probability of answering “no” to almost all items, indicating that the group’s overall health was good, so class 1 was named “relatively healthy.” Conversely, class 2 had the highest probability of answering “yes” to most of the items, indicating that the group’s overall health status was poor, so class 2 was named “multi-frail”. The conditional probabilities of class 3, class 4 and class 5 answering “yes” to most of the items were between those of class 1 and class 2. Class 3 had a higher probability of performing poorly on items representing functional activities (items 16–21) and cognitive function (item 33), so class 3 was named “cognitive and functionally frail”. Class 4 had a higher probability of performing poorly on items representing mental state (items 26–31), so class 4 was named “psychologically frail”, and this group had the highest probability of physical pain and poor sleep. Class 5 was named “physiologically frail”, because the health problems of this group were mainly concentrated on the components representing general health status and symptoms.

The number of elderly people in the five classes was 2,817 (52.7%), 176 (3.3%), 508 (9.5%), 638 (11.9%) and 1,202 (22.5%), respectively. The FI score of all participants was calculated. The “relatively healthy” group had the lowest average FI score (0.07±0.04), the “multi-frail” group had the largest (0.61±0.15), and the average FI scores of the “cognitive and functionally frail” group, “psychologically frail” group and “physiologically frail” group were all between those of the above two groups.

### Analysis of factors associated with different frailty subtypes

Multinomial logistic regression was used to evaluate the associations between the 12 baseline characteristics and the frailty subtypes. “Relatively healthy” was used as a reference class for the other four classes.

As shown in Table 3, the results showed that compared with people in the 60–70-year age group, the risk of having one of the four frailty subtypes in over 80-year-olds was higher. Elderly individuals who had not received education were also more likely to have one of the frailty subtypes than those who had received junior high school education or above. Those who were widowed, divorced or unmarried were more likely to be multi-frail, cognitive and functionally frail and psychologically frail compared to those who were married, and males had a lower risk of being cognitive and functionally frail and psychologically frail than females. Elderly people who had harmonious relationships with their family had a lower risk of being multi-frail than others; the fewer times the elderly were visited by their children, the more likely they were to be psychologically frail. The elderly who were dissatisfied with their housing had a higher risk of being cognitive and functionally frail, psychologically frail and physiologically frail, and those for whom pensions were their main source of income had a higher risk of being cognitive and functionally frail and psychologically frail than others. Individuals who exercised every day were less likely to be multi-frail and cognitive and functionally frail than those who never exercised, and those with a lower level of physical activity were more likely to have one of the four frailty subtypes. However, number of children and smoking status showed no significant effect on any of the frailty subtypes.

**Table 3:** Multinomial logistic regression analysis result for association of frailty subtypes with factors^@^

**Frailty subtypes**	**Variables**	**Walds**	***P value***	**OR**	**95% CI**
Multi-frail	**Intercept**	30.57	<0.001		
	**Age**				
	80~	24.07	<0.001	6.43	3.06~13.51
	60~*	.	.	1	.
	**Educational** **level**				
	Illiteracy	4.25	0.04	2.59	1.05~6.41
	Junior high school and above	.	.	1	.
	**Marital status**				
	Widowed / Divorced / Unmarried	16.87	<0.001	2.88	1.74~4.76
	Married*	.	.	1	.
	**Relationship with family**				
	Harmonious	11.41	0.001	0.21	0.08~0.52
	Not harmonious*	.	.	1	.
	**Exercise frequency**				
	Every day	11.30	0.001	0.39	0.23~0.68
	Never exercised*	.	.	1	.
	**Physical activity level**				
	Low level	52.02	<0.001	35.30	13.40~92.98
	Medium level	10.26	0.001	4.94	1.86~13.12
	High level*	.	.	1	.
Cognitive and functionally frail	Intercept	35.34	<0.001		
	**Age**				
	80~	127.11	<0.001	11.72	7.64~17.98
	70~	21.14	<0.001	2.28	1.60~3.23
	60~*	.	.	1	.
	**Sex**				
	Male	8.63	0.003	0.59	0.42~0.84
	Female*	.	.	1	.
	**Educational level**				
	Illiteracy	6.47	0.01	1.86	1.15~3.01
	Junior high school and above	.	.	1	.
	**Marital status**				
	Widowed / Divorced / Unmarried	12.21	<0.001	1.67	1.25~2.22
	Married*	.	.	1	.
	**Housing satisfaction**				
	Dissatisfied	4.80	0.03	2.93	1.12~7.66
	Satisfied*	.	.	1	.
	**Whether pensions are the main source of income**				
	Yes	13.20	<0.001	1.58	1.24~2.03
	No*	.	.	1	.
	**Exercise frequency**				
	Every day	20.18	<0.001	0.51	0.38~0.68
	Never exercised*	.	.	1	.
	**Physical activity level**				
	Low level	39.69	<0.001	3.35	2.30~4.88
	Medium level	12.55	<0.001	1.73	1.28~2.35
	High level*	.	.	1	.
Psychologically frail	Intercept	34.12	<0.001		
	**Age**				
	80~	6.34	0.01	1.64	1.12~2.41
	60~*	.	.	1	.
	**Sex**				
	Male	9.76	0.002	0.64	0.48~0.84
	Female*	.	.	1	.
	**Educational level**				
	Illiteracy	4.13	0.04	1.48	1.01~2.15
	Junior high school and above*	.	.	1	.
	**Marital status**				
	Widowed / Divorced / Unmarried	18.84	<0.001	1.73	1.35~2.21
	Married*	.	.	1	.
	**The number of times children visit per month**				
	0~2	27.07	<0.001	2.99	1.98~4.51
	3~4	13.45	<0.001	1.99	1.38~2.87
	5~*	.	.	1	.
	**Housing satisfaction**				
	Dissatisfied	19.90	<0.001	4.91	2.44~9.88
	General	20.70	<0.001	1.76	1.38~2.25
	Satisfied*	.	.	1	.
	**Whether pensions are the main source of income**				
	Yes	8.89	0.003	1.36	1.11~1.67
	No*	.	.	1	.
	**Physical activity level**				
	Medium level	14.25	<0.001	1.53	1.23~1.91
	High level*	.	.	1	.
Physiologically frail	Intercept	53.88	<0.001		
	**Age**				
	80~	13.51	<0.001	1.74	1.30~2.34
	70~	7.76	0.01	1.28	1.08~1.52
	60~*	.	.	1	.
	**Educational level**				
	Illiteracy	10.72	0.001	1.61	1.21~2.14
	Primary school	9.58	0.002	1.38	1.13~1.69
	Junior high school and above*	.	.	1	.
	**Housing satisfaction**				
	Dissatisfied	7.46	0.01	2.56	1.30~5.03
	General	28.03	<0.001	1.65	1.37~1.99
	Satisfied*	.	.	1	.
	**Physical activity level**				
	Medium level	87.52	<0.001	2.18	1.85~2.56
	High level*	.	.	1	.

^**@:**^ Multinomial logistic regression was used to evaluate the associations between the 12 baseline characteristics and the frailty subtypes. “Relatively healthy” was used as a reference class for the four frailty subtypes.

*Reference category of variables.

Abbreviations: OR, odds ratios; CI, confidence interval.

## DISCUSSION

To the best of our knowledge, this is the first study to explore frailty subtypes among Chinese elderly people by applying a validated assessment tool containing multiple components. From latent class analysis of the different functional components, we identified four frailty subtypes. In addition, we identified risk factors associated with these subtypes, which could provide a reference for targeted health intervention strategies for frail elderly people.

Our results identified five subpopulations within the population of older people and four frailty subtypes. The proportion of the population who were “relatively healthy” was 52.7%. The total proportion of relatively unhealthy individuals, including multi-frail, cognitive and functionally frail, psychologically frail and physiologically frail was 47.3%, which was similar to the prevalence of pre-frailty and frailty found in another elderly Chinese population [23]. This showed that a considerable proportion of elderly people are affected by different types and degrees of frailty. The emergence of frailty subtypes may reveal specific patterns of underlying problems in different domains, which in turn could help targeted interventions.

Among the five latent classes in this study, the “relatively healthy” group was least likely to have health problems on all components. The occurrence of a healthy group is consistent with previous research on frailty [13, 14, 24]. The FI (0.07±0.04) of this subpopulation was consistent with the past cutoff criteria for healthy groups (FI<0.08) [25]. Therefore, this group was naturally used as a control group for the other relatively unhealthy groups. The remaining “relatively unhealthy” older people were divided into four smaller classes of frailty.

Older people labeled as multi-frail had the worst health status and had health problems involving all components. This subpopulation had the highest probability of response on 26/33 health issues, and they had the highest score on the frailty index (0.61±0.15). In particular, this group was the only group that had health problems in “activities of daily living” and they needed help with almost all activities, i.e., they could be described as totally dependent. The proportion of the sample population that were classed as multi-frail was not insignificant, 3.3.%. Therefore, it is an urgent requirement to reduce the occurrence of this frailty subtype and provide reasonable health services for this subpopulation, to reduce the burden on families and society.

People in the “cognitive and functionally frail” group reported that health problems involving functional activities and cognitive function were much more severe than those in the “psychologically frail” and “physiologically frail” groups, second only to the “multi-frail”. In previous studies, the operational definition of cognitive frailty (CF) has been proposed as the co-existence of mild cognitive impairment (MCI) and physical frailty that is usually defined by Fried’s criteria, including unintentional weight loss, exhaustion, weakness, slowness, and inactivity [18, 26]. These five health issues were not exactly the same as the functional components mentioned in our study. Cognitive decline has been identified as a potent risk factor for functional decline [27]. Therefore, “cognitive and functionally frail” may be a new subtype, or it may be an extension of the previous definition of CF. Further research on the relationships among cognitive impairment, physical frailty, and functional activities may promote the emergence of a more comprehensive operational definition.

In the “psychologically frail” group, individuals experienced problems in relation to their mental state. In particular, among all subtypes, the probability of physical pain and poor sleep was highest in this group. In a previous study that examined psychologically frail elderly persons, the author suggested that people in this group were sensitive and regarded every inconvenience as a major problem. Indeed, in some countries, psychological problems among elderly individuals can seriously affect quality of life and has become a public health challenge [28]. Our results showed that there may be an interaction between mental state, body pain, and poor sleep, consistent with previous studies. One study pointed out that poor sleep was associated with severity of chronic pain [29]. Some studies have found that sleeping time is negatively correlated with psychological distress [28]. Another study reported that moderate pain was related to frailty in those older than 65 years; besides the direct influence of pain on frailty, depression was shown to partially mediate the pain-frailty nexus [34].

Participants classed as physiological frail were those who primarily suffered from problems in the general health status and symptoms domains. The elderly in this subtype had the highest probability of chronic diseases, visual impairment and hearing impairment. As far as we are aware, this subtype has not been identified elsewhere in the literature. Physical frailty has been mentioned in previous studies; however, there are key differences between the subtypes. Physiological frailty emphasizes the patient’s objective symptoms, while physical frailty emphasizes the lack of independence of the patient’s physical activity [14].

This study highlights the complexity of frailty and reflects the different underlying problems of the frail population, which are not well reflected by traditional frailty indexes. Although their FI scores were above the cutoff value of the frail group (FI ≥ 0.22) [25], older people in the multi-frail and cognitive and functionally frail groups experienced rather different problems. In the cognitive and functionally frail group, the problems mostly originated in the cognitive and functional domains, whereas people in the multi-frail group suffered from a combination of problems in all domains. In addition, the psychologically frail and physiologically frail groups had similar FI scores and they should both be classified as pre-frail based on the cutoff value of FI (0.08≤FI<0.22) [25], but the underlying problems clearly differed. Our results also found that the health problems of each domain did not emerge in separate subpopulations, which provides valuable insight into the complex interaction of health problems in the frail elderly.

With regard to factors associated with frailty subtypes, the results of the multivariate analysis showed that old age and low education level were the common risk factors among the four subtypes. This result is consistent with most previous studies in which age and educational level were identified as factors influencing frailty [30, 31]. Being widowed, divorced or unmarried was a risk factor for being multi-frail, cognitive and functionally frail and psychologically frail in the elderly but showed no effect on physiological frailty. This may be linked to the possibility that older persons who live with their spouses may receive emotional, economic and social support [32]. However, the influence of the spouse may not be significant in the physiological domain. In addition, older males had a lower risk of suffering cognitive and functional frailty and psychologically frailty than their female counterparts. This is consistent with previous studies, in which being female was found to be a risk factor for cognitive impairment and psychological distress [33, 34].

Having a harmonious relationship with family was a protective factor against multi-frailty. The fewer times the elderly were visited by their children per month, the higher the risk of suffering psychological frailty. Few studies have reported the impact of family relationships on frailty, but our result may be due to the influence of family relationships on psychological components. A study by Kawamoto found that family relationship was an explanatory variable for mental health in community-dwelling older persons [33]. Similarly, a study by Suwanmanee found that elderly people with a good family relationship had 4.9 times better mental health than those without a good family relationship [34]. In addition, visits by children to the elderly may bring psychological comfort, especially for those who do not have a partner. After all, family support, especially the support of children, plays a very important role in improving the health of elderly populations [35].

Dissatisfaction with housing was a risk factor for cognitive and functional frailty, psychological frailty and physiological frailty, and having a pension as the main source of income was a risk factor for cognitive and functional frailty and psychologically frailty. Housing satisfaction and whether a pension is the main source of income could reflect the economic situation of older people. In many studies, poor economic status has been considered a risk factor for frailty in the elderly [7, 36]. From another perspective, satisfaction with housing may be a reflection of the housing conditions of the elderly. A related study reported that poor housing conditions were independently associated with limitations in physical function and frailty in older adults [37].

Exercising every day was a protective factor against multi-frailty and cognitive and functional frailty, and a lower level of physical activity in the elderly was a risk factor for all four frailty subtypes. The regular practice of physical exercise is a simple and inexpensive way of preventing and treating various illnesses in elderly people [38]. The benefit of exercise and physical activity in aging populations, particularly with regard to frailty, has been proven in recent research; for example, a randomized multicenter controlled trial reported that an innovative multicomponent exercise program had benefits for functional and cognitive status among pre-frail/frail patients with mild cognitive impairment or dementia. Low physical activity has been found to be associated with frailty or pre-frailty in some prospective cohort studies [20, 39].

### Limitations and strengths

The main strength of this study is our consideration of the heterogeneity of frailty in the analysis. We expanded the commonly used model of frailty deficit accumulation, combined categorical variables with latent class models, and explored the interactions between functional components using latent class analysis. Our discovery of four frailty subtypes verified the complexity and heterogeneity of frailty. We further found that several frailty subtypes had different associated risk factors, which implies that targeted management and care interventions are required. Another strength of this study was the choice of assessment tool, which was well suited to the study participants. Considering the geographical and cultural differences between China and other countries, when compared with the translated version of the foreign scale, the FI scale developed by the project team and used in this study was more suitable for the Chinese elderly population. Finally, the large sample, combined with the full range of domains of functioning made the current research and discoveries valuable.

Our research also inevitably had some limitations. First, LCA is a person-centered approach to identify unobserved groups of similar individuals [14]. The selected research population may have an impact on the final latent classes. Although we conducted tracked home visits and telephone interviews for elderly people who were not able to participate in the medical examination, there were inevitably some elderly people who refused to be interviewed for various reasons. It is unclear whether the absence of these individuals affected the final results. Second, our research was only an exploration of frailty subtypes, it did not provide an operational definition for each subtype, and the validity of these subtypes requires further testing. Finally, frailty is a dynamic process [40]; our cross-sectional analysis did not include frailty trajectories. In this regard, it would be beneficial to explore whether a frailty subtype eventually transfers to another frailty subtype and how the trajectory of individuals progresses.

## MATERIALS AND METHODS

### Data and participants

We selected 21 communities (villages) in Dalang Town, Dongguan City, China, using a random cluster sampling method, and conducted cross-sectional surveys of all eligible seniors in these communities (villages). Elderly individuals aged 60 years and over and who were conscious were included; those diagnosed with Alzheimer's disease, a disability causing them to be bedridden, unable to communicate adequately, or unwilling to be investigated were excluded. The majority of participants were investigated during on-site physical examinations in Dalang Town from October 2017 to June 2019. In response to the national pension service policy, this medical examination was carried out free of charge for residents over 60 years old in all 28 communities (villages). We applied a questionnaire in the form of face-to-face interviews for the elderly who participated in the medical examination. In order to ensure the integrity of the sample, after completing the scheduled medical examinations, we immediately contacted all those elderly persons who did not present for the on-site physical examination. We conducted surveys face-to-face at these participants’ homes or via telephone.

A total of 5397 questionnaires were distributed. After excluding 56 unqualified questionnaires, 5341 samples were finally included in the analysis; the effective response rate was 98.96%.

### Composition of the questionnaire

### Frailty assessment tool

The FI scale developed by the project team at an early stage was used in this research, containing seven dimensions of a total of 33 health problems. General health status contained six items (e.g. Are you in poor health now? Has your health deteriorated compared to 1 year ago?) Activities of daily living contained nine items (e.g., In the past month, did you need help to complete the following activities? Bathing, Dressing, etc.). Functional activities contained six items (e.g. In the past month, did you need help to complete the following activities? Going up or down stairs, shopping, etc.). Symptoms contained four items (e.g. Have you had any physical pain in the past month? Is your vision impaired?). Mental state contained six items (e.g. Have you had the following feelings in the past week? Hard to concentrate; Feeling sad or depressed, etc.). Social support contained one item (Are you living alone?). For the above 32 questions, the respondent only needed to answer "Yes" or "No" according to their own circumstances. In addition, cognitive function was assessed using the Mini-Mental State Examination (MMSE). The investigator evaluated whether respondents’ cognitive function was poor based on their final scores. A score of 17 points or less was marked as "Yes", and a score of more than 17 points was marked as "No" [41, 42].

From our previous studies, the FI scale developed was based on a frailty index model. After debugging among the elderly population in China, the scale has been shown to fully reflect the frailty condition of Chinese elderly, and its reliability and validity has been proven to be good [25]. The number of health problems (answering "yes") of each elderly person was divided by the total number of 33 health problems, to obtain the FI score; the score ranged from 0 to 1, with a higher score indicating a higher level of frailty.

### Baseline characteristics

A total of 12 variables of four aspects were included in the questionnaire. Sociodemographic characteristics: sex (male; female), age (60–; 70–; 80–), educational level (illiteracy; primary school; junior high school and above), marital status (married; widowed/divorced/ unmarried). Family relationship: number of children (0–1; 2–3; 4–), number of times children visit per month (0–2; 3–4; ≥5), relationship with family (harmonious; not harmonious). Economic status: whether pensions are the main source of income (yes; no), housing satisfaction (dissatisfied; general; satisfied). Behavioral factors: smoking status (yes; no), exercise frequency (every day; less than five times a week; less than 2 times a week; not exercising), and physical activity level (low level; medium level; high level), which was measured using the short version of the International Physical Activity Questionnaire (IPAQ).

### Statistical analysis

### Data preparation and descriptive analysis

EpiData 3.1 software (The EpiData Association, Odense, Denmark) was used to establish the database; Microsoft Excel 2013 version was used for data collation and error correction. We generated descriptive statistics for the total sample, giving frequencies and percentages for the sociodemographic variables.

### Latent class analysis (LCA)

First, we used latent class analysis (LCA) to explore the latent classes of frailty. LCA is a statistical technique for exploring the categorical latent variables behind the statistically related categorical observed variables; it combines latent variable theory with categorical variables. Based on the observed variables, the purpose of LCA is to find the best class solution, that is, to explain the association among a set of observed variables with the least number of latent classes, and further to cluster similar individuals [43]. The observed variables we used in the LCA were the 33 variables mentioned above for the frailty assessment.

In this study, the maximum likelihood (ML) method was used for parameter estimation, and the expectation-maximum (EM) method was used in the iterative process. We fitted 1–7 latent class models. For model evaluation, the indicators included the Akaike information criterion (AIC), Bayesian information criterion (BIC), and adjusted BIC (aBIC). In general, the smaller the numerical values of these indicators, the better the model fits. The entropy index was used to evaluate the accuracy of the classification. A value greater than or equal to 0.80 in this index shows an accuracy of classification of greater than 90% [44]. The Lo–Mendell–Rubin (LMR) and the bootstrap-based likelihood ratio test (BLRT) were used to compare the differences in fit of the nested latent class models; if the *P* values of the two values achieved a significant level (*P* < 0.01), this indicated that the model with K classes was significantly better than the model with K–1 classes [45]. We determined the final classification on the basis of comprehensive consideration of the above indicators. The LCA analysis was done using Mplus7.4.

### Naming of latent classes

First, based on the final classes selected, the probability of each latent class and the conditional probability of each observed variable under the latent classes were estimated by the EM method. Next, according to the differences in the conditional probability distribution and characteristics of the observed variables, we interpreted and named each latent class. Finally, we calculated the FI score of each individual and described the FI score distribution $\left(\bar{x}\pm s\right)$ for each class.

### Analysis of factors associated with different frailty subtypes

In order to explore the factors associated with different frailty subtypes (latent classes), we conducted a multinomial logistic regression with frailty type of the elderly as the dependent variable and 12 possible associated variables as the independent variables. We used one category of each independent variable as a reference category to obtain the adjusted odds ratios (OR) and their 95% confidence intervals (CI) for the other categories; *P* ≤ 0.05 was considered statistically significant. This analysis was conducted using SPSS software (IBM SPSS Statistics 25.0, IBM Corporation).

### Ethics approval

Informed written consent was obtained from all participants included in this study. The study was approved by the Research Ethics Committee of Guangdong Medical University (YJYS2018046).

## Supplementary Material

Supplementary Table 1

## References

[1] Bottiger BA, Nicoara A, Snyder LD, Wischmeyer PE, Schroder JN, Patel CB, Daneshmand MA, Sladen RN, Ghadimi K. Frailty in the End-Stage Lung Disease or Heart Failure Patient: Implications for the Perioperative Transplant Clinician. J Cardiothorac Vasc Anesth. 2019; 33:1382–92. 10.1053/j.jvca.2018.08.002 30193783 PMC6368901

[2] Summers MJ, Rainero I, Vercelli AE, Aumayr G, de Rosario H, Mönter M, Kawashima R, My AH, and My-AHA Consortium. The My Active and Healthy Aging (My-AHA) ICT platform to detect and prevent frailty in older adults: randomized control trial design and protocol. Alzheimers Dement (N Y). 2018; 4:252–62. 10.1016/j.trci.2018.06.004 30094329 PMC6076210

[3] Dent E, Hoogendijk EO. Psychosocial factors modify the association of frailty with adverse outcomes: a prospective study of hospitalised older people. BMC Geriatr. 2014; 14:108. 10.1186/1471-2318-14-108 25262425 PMC4190287

[4] Chiou JH, Liu LK, Lee WJ, Peng LN, Chen LK. What factors mediate the inter-relationship between frailty and pain in cognitively and functionally sound older adults? A prospective longitudinal ageing cohort study in Taiwan. BMJ Open. 2018; 8:e018716. 10.1136/bmjopen-2017-018716 29453297 PMC5829604

[5] Carneiro JA, Cardoso RR, Durães MS, Guedes MC, Santos FL, Costa FM, Caldeira AP. Frailty in the elderly: prevalence and associated factors. Rev Bras Enferm. 2017; 70:747–52. 10.1590/0034-7167-2016-0633 28793104

[6] Ottenbacher KJ, Graham JE, Al Snih S, Raji M, Samper-Ternent R, Ostir GV, Markides KS. Mexican Americans and frailty: findings from the Hispanic established populations epidemiologic studies of the elderly. Am J Public Health. 2009; 99:673–79. 10.2105/AJPH.2008.143958 19197079 PMC2661466

[7] Yang F, Chen QW. Evaluation of frailty and influencing factors in old people in hospital institution: evidence for a phenotype of frailty. Medicine (Baltimore). 2018; 97:e9634. 10.1097/MD.0000000000009634 29504994 PMC5779763

[8] Hendry A, Carriazo AM, Vanhecke E, Rodríguez-Laso Á, Package AJ, and ADVANTAGE JA Work Package 7. Integrated Care: A Collaborative ADVANTAGE for Frailty. Int J Integr Care. 2018; 18:1. 10.5334/ijic.4156 30127685 PMC6095052

[9] Iqbal J, Denvir M, Gunn J. Frailty assessment in elderly people. Lancet. 2013; 381:1985–86. 10.1016/S0140-6736(13)61203-9 23746900

[10] Rockwood K, Mitnitski A. Frailty in relation to the accumulation of deficits. J Gerontol A Biol Sci Med Sci. 2007; 62:722–27. 10.1093/gerona/62.7.722 17634318

[11] Chamberlain AM, St Sauver JL, Jacobson DJ, Manemann SM, Fan C, Roger VL, Yawn BP, Finney Rutten LJ. Social and behavioural factors associated with frailty trajectories in a population-based cohort of older adults. BMJ Open. 2016; 6:e011410. 10.1136/bmjopen-2016-011410 27235302 PMC4885446

[12] Asada Y, Hurley J, Grignon M, Kirkland S. Health inequalities and inequities by age: Stability for the Health Utilities Index and divergence for the Frailty Index. SSM Popul Health. 2018; 5:17–32. 10.1016/j.ssmph.2018.04.002 30069499 PMC6066476

[13] Liu LK, Guo CY, Lee WJ, Chen LY, Hwang AC, Lin MH, Peng LN, Chen LK, Liang KY. Subtypes of physical frailty: latent class analysis and associations with clinical characteristics and outcomes. Sci Rep. 2017; 7:46417. 10.1038/srep46417 28397814 PMC5387710

[14] Looman WM, Fabbricotti IN, Blom JW, Jansen AP, Lutomski JE, Metzelthin SF, Huijsman R, and TOPICS-MDS Research Consortium. The frail older person does not exist: development of frailty profiles with latent class analysis. BMC Geriatr. 2018; 18:84. 10.1186/s12877-018-0776-5 29618334 PMC5885355

[15] Chng GS, Li D, Chu CM, Ong T, Lim F. Family profiles of maltreated children in Singapore: A latent class analysis. Child Abuse Negl. 2018; 79:465–75. 10.1016/j.chiabu.2018.02.029 29547839

[16] Ji Y, Xu H, Zhang Y, Liu Q. Heterogeneity of adolescent health risk behaviors in rural western China: A latent class analysis. PLoS One. 2018; 13:e0199286. 10.1371/journal.pone.0199286 29944679 PMC6019255

[17] Zhang Z, Abarda A, Contractor AA, Wang J, Dayton CM. Exploring heterogeneity in clinical trials with latent class analysis. Ann Transl Med. 2018; 6:119. 10.21037/atm.2018.01.24 29955579 PMC6015948

[18] Lee WJ, Peng LN, Liang CK, Loh CH, Chen LK. Cognitive frailty predicting all-cause mortality among community-living older adults in Taiwan: A 4-year nationwide population-based cohort study. PLoS One. 2018; 13:e0200447. 10.1371/journal.pone.0200447 30001354 PMC6042743

[19] Mulasso A, Roppolo M, Giannotta F, Rabaglietti E. Associations of frailty and psychosocial factors with autonomy in daily activities: a cross-sectional study in Italian community-dwelling older adults. Clin Interv Aging. 2016; 11:37–45. 10.2147/CIA.S95162 26811675 PMC4714730

[20] Bouillon K, Kivimäki M, Hamer M, Shipley MJ, Akbaraly TN, Tabak A, Singh-Manoux A, Batty GD. Diabetes risk factors, diabetes risk algorithms, and the prediction of future frailty: the Whitehall II prospective cohort study. J Am Med Dir Assoc. 2013; 14:851.e1–6. 10.1016/j.jamda.2013.08.016 24103860 PMC3820037

[21] Gale CR, Westbury L, Cooper C. Social isolation and loneliness as risk factors for the progression of frailty: the English Longitudinal Study of Ageing. Age Ageing. 2018; 47:392–97. 10.1093/ageing/afx188 29309502 PMC5920346

[22] Feng Z, Lugtenberg M, Franse C, Fang X, Hu S, Jin C, Raat H. Risk factors and protective factors associated with incident or increase of frailty among community-dwelling older adults: A systematic review of longitudinal studies. PLoS One. 2017; 12:e0178383. 10.1371/journal.pone.0178383 28617837 PMC5472269

[23] Ye B, Gao J, Fu H. Associations between lifestyle, physical and social environments and frailty among Chinese older people: a multilevel analysis. BMC Geriatr. 2018; 18:314. 10.1186/s12877-018-0982-1 30547760 PMC6295038

[24] Sousa-Santos AR, Afonso C, Santos A, Borges N, Moreira P, Padrão P, Fonseca I, Amaral TF. The association between 25(OH)D levels, frailty status and obesity indices in older adults. PLoS One. 2018; 13:e0198650. 10.1371/journal.pone.0198650 30153256 PMC6112621

[25] Zhang Y, Liang Y, Sun M, Huang L, Lian T, Huang J, Ni J. Analysis on influencing factors of the frailty of the elderly. Chinese Journal of Disease Control and Prevention (Chinese). 2019; 23:140–45.

[26] Ma L, Zhang L, Sun F, Li Y, Tang Z. Cognitive function in Prefrail and frail community-dwelling older adults in China. BMC Geriatr. 2019; 19:53. 10.1186/s12877-019-1056-8 30813907 PMC6391822

[27] Malek Rivan NF, Shahar S, Rajab NF, Singh DK, Din NC, Hazlina M, Hamid TA. Cognitive frailty among Malaysian older adults: baseline findings from the LRGS TUA cohort study. Clin Interv Aging. 2019; 14:1343–52. 10.2147/CIA.S211027 31413555 PMC6663036

[28] Yamamoto Y, Suzuki H, Owari Y, Miyatake N. Relationships between Physical Activity, Sleeping Time, and Psychological Distress in Community-Dwelling Elderly Japanese. Medicina (Kaunas). 2019; 55:318. 10.3390/medicina55070318 31252680 PMC6681241

[29] van Oostrom SH, van der A DL, Rietman ML, Picavet HS, Lette M, Verschuren WM, de Bruin SR, Spijkerman AM. A four-domain approach of frailty explored in the Doetinchem Cohort Study. BMC Geriatr. 2017; 17:196. 10.1186/s12877-017-0595-0 28854882 PMC5577839

[30] Lee DR, Santo EC, Lo JC, Ritterman Weintraub ML, Patton M, Gordon NP. Understanding functional and social risk characteristics of frail older adults: a cross-sectional survey study. BMC Fam Pract. 2018; 19:170. 10.1186/s12875-018-0851-1 30340530 PMC6195739

[31] Erlandson KM, Wu K, Koletar SL, Kalayjian RC, Ellis RJ, Taiwo B, Palella FJ Jr, Tassiopoulos K. Association Between Frailty and Components of the Frailty Phenotype With Modifiable Risk Factors and Antiretroviral Therapy. J Infect Dis. 2017; 215:933–37. 10.1093/infdis/jix063 28453849 PMC5407051

[32] Yang L, Jiang Y, Xu S, Bao L, Parker D, Xu X, Li J. Evaluation of frailty status among older people living in urban communities by Edmonton Frail Scale in Wuhu, China: a cross-sectional study. Contemp Nurse. 2018; 54:630–39. 10.1080/10376178.2018.1552525 30479179

[33] Kawamoto R, Doi T, Okayama M, Satho M, Kajii E. [A study of mental health in community-dwelling older persons—influence of cared elderly]. Nihon Ronen Igakkai Zasshi. 2000; 37:912–20. 10.3143/geriatrics.37.912 11193369

[34] Suwanmanee S, Nanthamongkolchai S, Munsawaengsub C, Taechaboonsermsak P. Factors influencing the mental health of the elderly in Songkhla, Thailand. J Med Assoc Thai. 2012; 95:S8–15. 23130483

[35] Luo H, Wu K, Qian J, Cao P, Ren XH. [Urban-Rural Differences in Family Support for the Physical and Mental Health of the Elderly in China.] Sichuan Da Xue Xue Bao Yi Xue Ban. 2017; 48:263–67. 28612539

[36] Gesualdo GD, Zazzetta MS, Say KG, Orlandi FS. [Factors associated with the frailty of elderly people with chronic kidney disease on hemodialysis]. Cien Saude Colet. 2016; 21:3493–98. 10.1590/1413-812320152111.18222015 27828582

[37] García-Esquinas E, Pérez-Hernández B, Guallar-Castillón P, Banegas JR, Ayuso-Mateos JL, Rodríguez-Artalejo F. Housing conditions and limitations in physical function among older adults. J Epidemiol Community Health. 2016; 70:954–60. 10.1136/jech-2016-207183 27225681

[38] Casas-Herrero A, Anton-Rodrigo I, Zambom-Ferraresi F, Sáez de Asteasu ML, Martinez-Velilla N, Elexpuru-Estomba J, Marin-Epelde I, Ramon-Espinoza F, Petidier-Torregrosa R, Sanchez-Sanchez JL, Ibañez B, Izquierdo M. Effect of a multicomponent exercise programme (VIVIFRAIL) on functional capacity in frail community elders with cognitive decline: study protocol for a randomized multicentre control trial. Trials. 2019; 20:362. 10.1186/s13063-019-3426-0 31208471 PMC6580555

[39] Brunner EJ, Shipley MJ, Ahmadi-Abhari S, Valencia Hernandez C, Abell JG, Singh-Manoux A, Kawachi I, Kivimaki M. Midlife contributors to socioeconomic differences in frailty during later life: a prospective cohort study. Lancet Public Health. 2018; 3:e313–22. 10.1016/S2468-2667(18)30079-3 29908857 PMC6120440

[40] Gobbens RJ, Luijkx KG, Wijnen-Sponselee MT, Schols JM. Towards an integral conceptual model of frailty. J Nutr Health Aging. 2010; 14:175–81. 10.1007/s12603-009-0142-620191249

[41] Kutschar P, Weichbold M, Osterbrink J. Effects of age and cognitive function on data quality of standardized surveys in nursing home populations. BMC Geriatr. 2019; 19:244. 10.1186/s12877-019-1258-0 31481012 PMC6724313

[42] Lu H, Wang XD, Shi Z, Yue W, Zhang Y, Liu S, Liu S, Zhao L, Xiang L, Zhang Y, Guan Y, Su W, Li Z, et al. Comparative analysis of cognitive impairment prevalence and its etiological subtypes in a rural area of northern China between 2010 and 2015. Sci Rep. 2019; 9:851. 10.1038/s41598-018-37286-z 30696930 PMC6351643

[43] Muthén B, Muthén LK. Integrating person-centered and variable-centered analyses: growth mixture modeling with latent trajectory classes. Alcohol Clin Exp Res. 2000; 24:882–91. 10.1111/j.1530-0277.2000.tb02070.x 10888079

[44] Seymour J, McNamee P, Scott A, Tinelli M. Shedding new light onto the ceiling and floor? A quantile regression approach to compare EQ-5D and SF-6D responses. Health Econ. 2010; 19:683–96. 10.1002/hec.1505 19504545

[45] Muthén B, Asparouhov T. Latent variable analysis with categorical outcomes: Multiple-group and growth modeling in Mplus. Mplus Web Notes. 2002; 4:1–22. https://www.statmodel.com/download/webnotes/CatMGLong.pdf

