# Supplementary material for: Development of frailty subtypes and their associated risk factors among the community-dwelling elderly population
Source: Aging (Albany NY). 2020 Jan 16;12(2):1128–40. doi: 10.18632/aging.102671 (PMC7053645; doi:10.18632/aging.102671)
Supplement: Supplementary Table 1 [file aging-12-102671-s001..docx]

**Supplementary Table 1. Conditional probability distribution of observed variables under the five latent classes.**

| **Items** | **Class1** | **Class2** | **Class3** | **Class4** | **Class5** |
| --- | --- | --- | --- | --- | --- |
| **General health status** |  |  |  |  |  |
| 1.Are you in poor health now? |  |  |  |  |  |
| No | 0.475 | 0.118 | 0.190 | 0.131 | 0.303 |
| Yes | 0.525 | 0.882 | 0.810 | 0.869 | 0.697 |
| 2. Has your health deteriorated compared to 1 year ago? |  |  |  |  |  |
| No | 0.900 | 0.497 | 0.635 | 0.626 | 0.709 |
| Yes | 0.100 | 0.503 | 0.365 | 0.374 | 0.291 |
| 3. Have you been to the hospital in the past year? |  |  |  |  |  |
| No | 0.751 | 0.435 | 0.572 | 0.564 | 0.528 |
| Yes | 0.249 | 0.565 | 0.428 | 0.436 | 0.472 |
| 4. Did you fall in the past year? |  |  |  |  |  |
| No | 0.946 | 0.707 | 0.808 | 0.862 | 0.864 |
| Yes | 0.054 | 0.293 | 0.192 | 0.138 | 0.136 |
| 5. Is the BMI at normal level? |  |  |  |  |  |
| No | 0.571 | 0.527 | 0.586 | 0.633 | 0.533 |
| Yes | 0.429 | 0.473 | 0.414 | 0.367 | 0.467 |
| 6. Do you have any chronic diseases now? |  |  |  |  |  |
| No | 0.476 | 0.232 | 0.288 | 0.339 | 0.228 |
| Yes | 0.524 | 0.768 | 0.712 | 0.661 | 0.772 |
| **Activities of daily living**  （In the past month, did you need help to complete the following activities?） |  |  |  |  |  |
| 7. Bathing |  |  |  |  |  |
| No | 1 | 0.073 | 0.986 | 1 | 1 |
| Yes | 0 | 0.927 | 0.014 | 0 | 0 |
| 8. Dressing |  |  |  |  |  |
| No | 1 | 0.169 | 0.996 | 1 | 1 |
| Yes | 0 | 0.831 | 0.004 | 0 | 0 |
| 9. Eating |  |  |  |  |  |
| No | 0.999 | 0.328 | 0.997 | 1 | 1 |
| Yes | 0.001 | 0.672 | 0.003 | 0 | 0 |
| 10. Cooking |  |  |  |  |  |
| No | 0.99 | 0.052 | 0.775 | 0.993 | 0.995 |
| Yes | 0.01 | 0.948 | 0.225 | 0.007 | 0.005 |
| 11. Washing |  |  |  |  |  |
| No | 0.999 | 0.036 | 0.855 | 0.995 | 0.996 |
| Yes | 0.001 | 0.964 | 0.145 | 0.005 | 0.004 |
| 12. Walking around the house |  |  |  |  |  |
| No | 0.999 | 0.099 | 0.95 | 1 | 1 |
| Yes | 0.001 | 0.901 | 0.05 | 0 | 0 |
| 13. Controlling defecation |  |  |  |  |  |
| No | 1 | 0.269 | 0.989 | 0.998 | 1 |
| Yes | 0 | 0.731 | 0.011 | 0.002 | 0 |
| 14. Toileting |  |  |  |  |  |
| No | 1 | 0.161 | 0.989 | 1 | 1 |
| Yes | 0 | 0.839 | 0.011 | 0 | 0 |
| 15. Dressing up |  |  |  |  |  |
| No | 1 | 0.256 | 0.978 | 0.997 | 1 |
| Yes | 0 | 0.744 | 0.022 | 0.003 | 0 |
| **Functional activity**  （In the past month, did you need help to complete the following activities?） |  |  |  |  |  |
| 16. Up or down stairs |  |  |  |  |  |
| No | 0.99 | 0.109 | 0.348 | 0.965 | 0.981 |
| Yes | 0.01 | 0.891 | 0.652 | 0.035 | 0.019 |
| 17. Shopping |  |  |  |  |  |
| No | 0.988 | 0.051 | 0.378 | 0.997 | 0.995 |
| Yes | 0.012 | 0.949 | 0.622 | 0.003 | 0.005 |
| 18. Calling |  |  |  |  |  |
| No | 0.978 | 0.107 | 0.389 | 0.973 | 0.951 |
| Yes | 0.022 | 0.893 | 0.611 | 0.027 | 0.049 |
| 19. Walking far distance |  |  |  |  |  |
| No | 0.994 | 0.08 | 0.199 | 0.986 | 0.997 |
| Yes | 0.006 | 0.92 | 0.801 | 0.014 | 0.003 |
| 20. Going out alone |  |  |  |  |  |
| No | 0.97 | 0.103 | 0.136 | 0.956 | 0.982 |
| Yes | 0.03 | 0.897 | 0.864 | 0.044 | 0.018 |
| 21. Stooping to pick up something on the ground |  |  |  |  |  |
| No | 0.941 | 0.157 | 0.179 | 0.845 | 0.95 |
| Yes | 0.059 | 0.843 | 0.821 | 0.155 | 0.05 |
| **Symptom** |  |  |  |  |  |
| 22. Have you had any physical pain in the past month? |  |  |  |  |  |
| No | 0.607 | 0.242 | 0.255 | 0.18 | 0.355 |
| Yes | 0.393 | 0.758 | 0.745 | 0.82 | 0.645 |
| 23. Is your vision impaired? |  |  |  |  |  |
| No | 0.904 | 0.436 | 0.616 | 0.602 | 0.225 |
| Yes | 0.096 | 0.564 | 0.384 | 0.398 | 0.775 |
| 24. Is your hearing impairment? |  |  |  |  |  |
| No | 0.957 | 0.526 | 0.693 | 0.73 | 0.398 |
| Yes | 0.043 | 0.474 | 0.307 | 0.27 | 0.602 |
| 25. Have you had a poor sleep in the past month? |  |  |  |  |  |
| No | 0.72 | 0.361 | 0.415 | 0.217 | 0.542 |
| Yes | 0.28 | 0.639 | 0.585 | 0.783 | 0.458 |
| **Mental state**  (Have you had the following feelings in the past week?) |  |  |  |  |  |
| 26. Hard to concentrate |  |  |  |  |  |
| No | 0.992 | 0.656 | 0.779 | 0.692 | 0.979 |
| Yes | 0.008 | 0.344 | 0.221 | 0.308 | 0.021 |
| 27. Feeling Sad or depressed |  |  |  |  |  |
| No | 0.939 | 0.428 | 0.687 | 0.256 | 0.88 |
| Yes | 0.061 | 0.572 | 0.313 | 0.744 | 0.12 |
| 28. Feeling lonely |  |  |  |  |  |
| No | 0.981 | 0.528 | 0.736 | 0.548 | 0.981 |
| Yes | 0.019 | 0.472 | 0.264 | 0.452 | 0.019 |
| 29. Memory was worse than peers |  |  |  |  |  |
| No | 0.901 | 0.489 | 0.638 | 0.425 | 0.524 |
| Yes | 0.099 | 0.511 | 0.362 | 0.575 | 0.476 |
| 30. Didn't like going out (connecting with people) |  |  |  |  |  |
| No | 0.969 | 0.645 | 0.817 | 0.786 | 0.93 |
| Yes | 0.031 | 0.355 | 0.183 | 0.214 | 0.07 |
| 31. Feeling tired and not spiritual |  |  |  |  |  |
| No | 0.985 | 0.595 | 0.822 | 0.702 | 0.929 |
| Yes | 0.015 | 0.405 | 0.178 | 0.298 | 0.071 |
| **Social support** |  |  |  |  |  |
| 32. Are you living alone. |  |  |  |  |  |
| No | 0.945 | 0.661 | 0.671 | 0.81 | 0.88 |
| Yes | 0.055 | 0.339 | 0.329 | 0.19 | 0.12 |
| **Cognitive function** |  |  |  |  |  |
| 33. Poor cognitive function |  |  |  |  |  |
| No | 0.948 | 0.362 | 0.528 | 0.852 | 0.887 |
| Yes | 0.052 | 0.638 | 0.472 | 0.148 | 0.113 |
| **Latent classes probability** | 0.527 | 0.033 | 0.095 | 0.119 | 0.225 |
| **Sample frequency** | 2817 | 176 | 508 | 638 | 1202 |
| **FI score（$\bar{x}\pm s$）** | 0.07±0.04 | 0.61±0.15 | 0.30±0.12 | 0.21±0.07 | 0.16±0.05 |

Abbreviations: BMI, body mass index; FI, frailty Index.
